# Supplementary material for: Insights into the Taxonomically Challenging Hexaploid Alpine Shrub Willows of Salix Sections Phylicifoliae and Nigricantes (Salicaceae)
Source: Plants (Basel). 2023 Mar 2;12(5):1144. doi: 10.3390/plants12051144 (PMC10005704; doi:10.3390/plants12051144)
Supplement: Supplementary file 1 [file plants-12-01144-s001.zip › Suppl_Fig_S1_RAxML_backbone_quartetsampling.pdf]

Wagner et al. 2023

Supplement Fig. S1 RAxML phylogeny including QS values

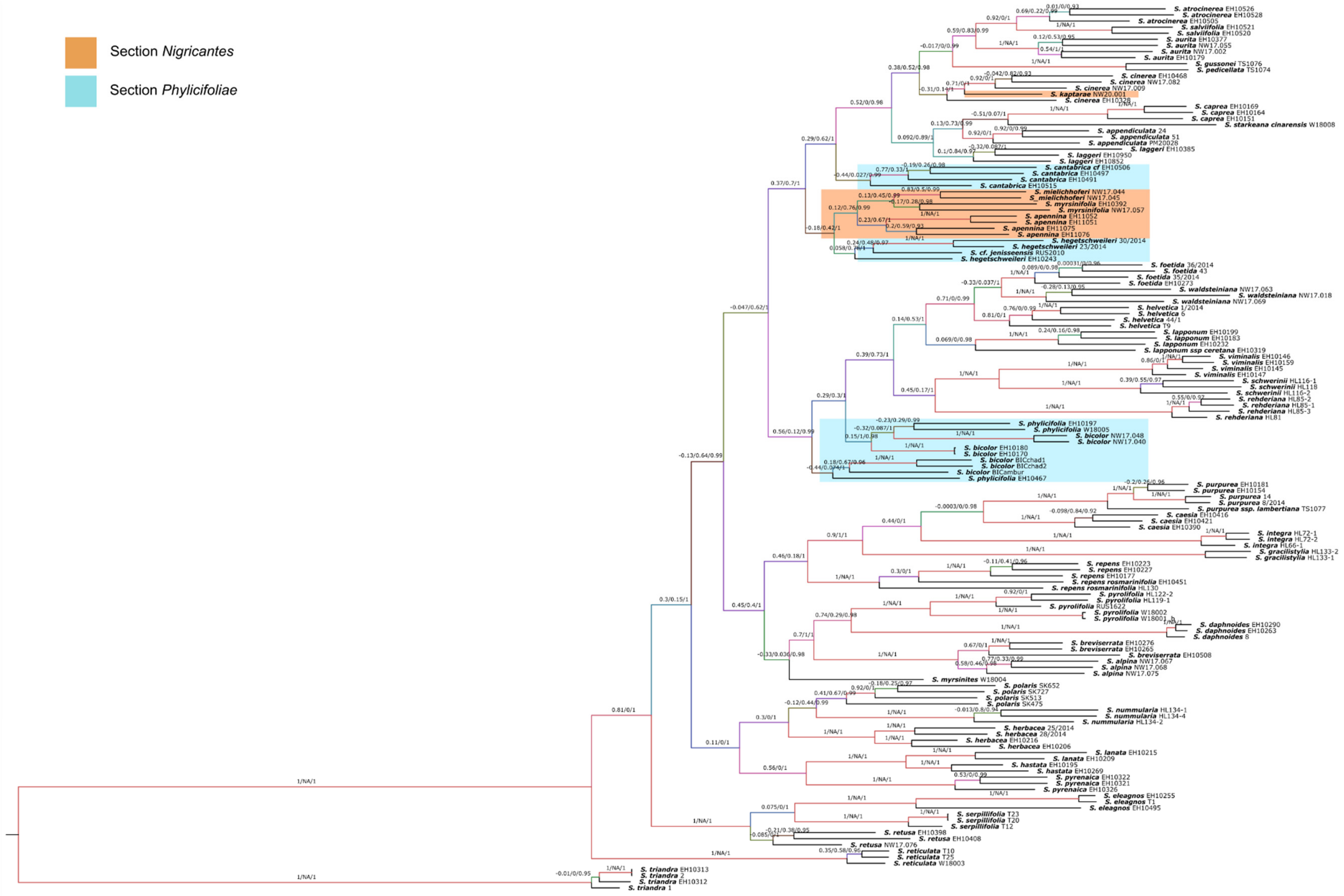

Fig S1 – Backbone topology of the *Chamaetia/Vetrix* clade including 145 samples representing 45 Eurasian species plus *S. triandra* as outgroup based on a RAxML analyses on 46,568 RAD loci (630,764 SNPs) in a concatenated alignment of 5,341,180 bp and 66.95% missing sites. Quartetsampling values above branches. Species of sections *Nigricantes* and *Phylicifoliae* are highlighted in colors according to legend.
