# Supplementary material for: Insights into the Taxonomically Challenging Hexaploid Alpine Shrub Willows of Salix Sections Phylicifoliae and Nigricantes (Salicaceae)
Source: Plants (Basel). 2023 Mar 2;12(5):1144. doi: 10.3390/plants12051144 (PMC10005704; doi:10.3390/plants12051144)
Supplement: Supplementary file 1 [file plants-12-01144-s001.zip › Suppl_Fig_S2_RAxML_hexaploids.pdf]

Supplement Fig. S2 - RAxML

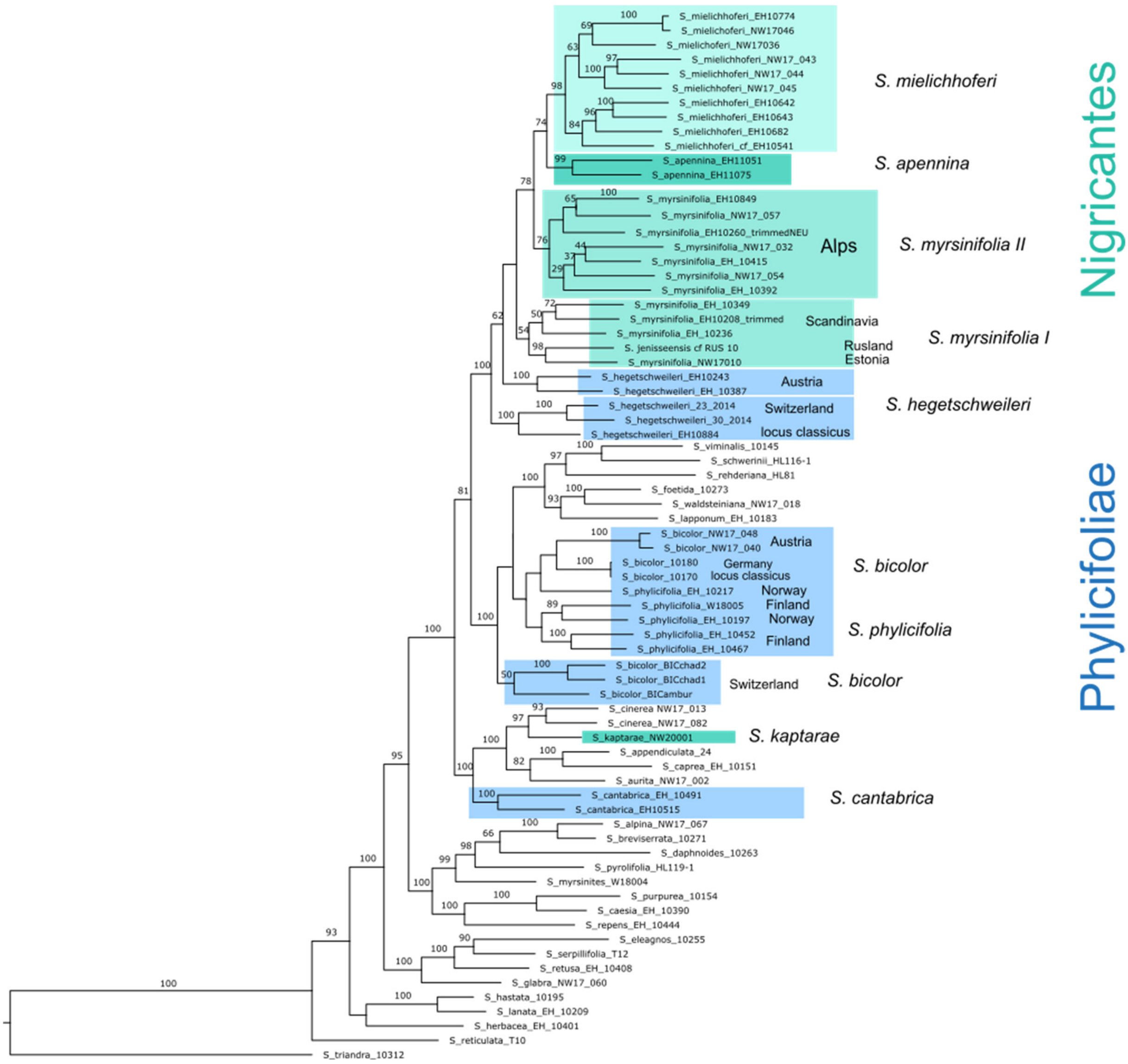

Fig. S2 RAxML analyses of all samples of the analysed species of sections *Nigricantes* (green) and *Phylicifoliae* (blue) with a reduced backbone sampling of taxa representing the *Chamaetia/Vetrix* clade. The dataset comprised 73 samples and was based on 29,395 RAD loci resulting in a concatenated alignment of 3,466,391 bp including 179,212 SNPs and 72.08% missing sites. Bootstrap values >50 above branches, sectional assignment color coded.

The ipyrad settings for this analysis was filtered by including only loci shared by minimum 10 individuals, show maximum four alleles, max. 10 SNPs per locus and a similarity threshold of 85%.
