# Supplementary material for: Insights into the Taxonomically Challenging Hexaploid Alpine Shrub Willows of Salix Sections Phylicifoliae and Nigricantes (Salicaceae)
Source: Plants (Basel). 2023 Mar 2;12(5):1144. doi: 10.3390/plants12051144 (PMC10005704; doi:10.3390/plants12051144)
Supplement: Supplementary file 1 [file plants-12-01144-s001.zip › Suppl_Fig_S3 clade wise RADpainter.pdf]

Supplement Fig S3 - Clade wise co-ancestry matrix for *Nigricantes* and *Phylicifoliae*

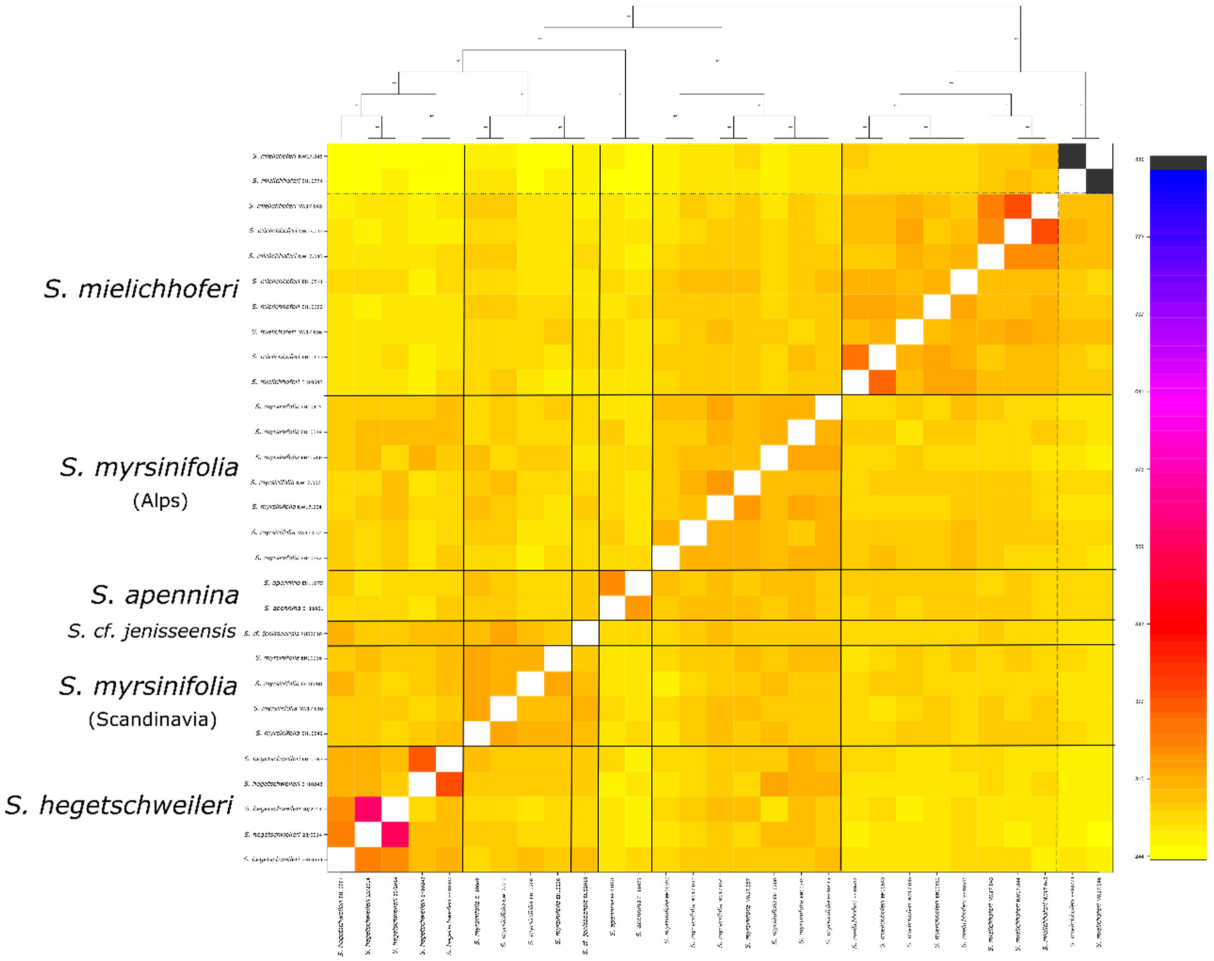

Fig S3 a. Co-ancestry matrix of the section *Nigricantes* clade based on 8,164 RADloci (17% missing data). The species *S. mielichhoferi* and *S. myrsinifolia* show levels of shared loci (co-ancestry; orange to red coloured squares). The samples of *S. hegetschweileri* and *S. myrsinifolia* also show levels of co-ancestry or hybridisation, respectively. *Salix cf. jenisseensis* shows a higher co-ancestry with Scandinavian *S. myrsinifolia* samples than with alpine samples.

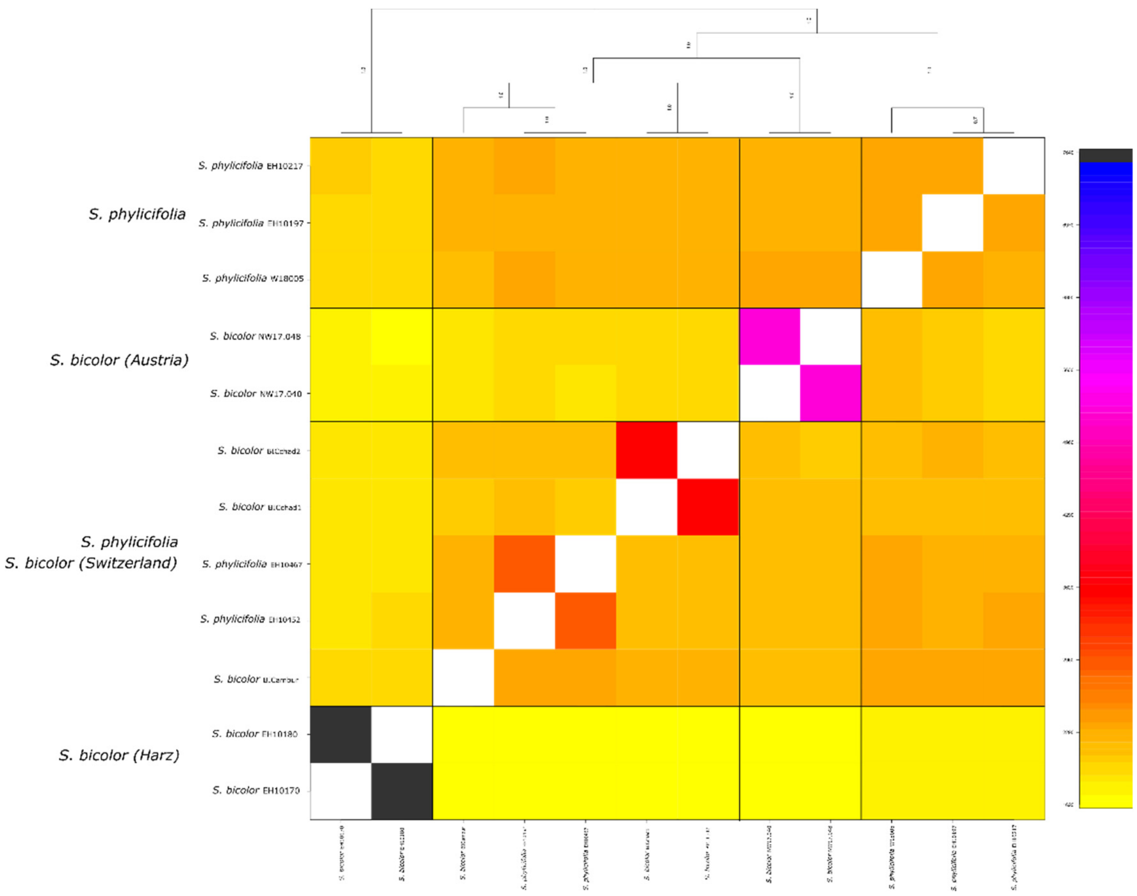

Fig S3 b. Co-ancestry matrix resulting from RADpainter based on 27,010 RAD loci (6.51% missing data) of *S. phylicifolia* and *S. bicolor*. The samples from Harz Mountain are separate and show very low levels of co-ancestry (yellow color). Triploid samples from Austria show only low levels of co-ancestry with the remaining samples, while hexaploid samples of *S. phylicifolia* and *S. bicolor* from Switzerland show high number of shared loci indicated by orange and red colors.
