# Supplementary material for: Insights into the Taxonomically Challenging Hexaploid Alpine Shrub Willows of Salix Sections Phylicifoliae and Nigricantes (Salicaceae)
Source: Plants (Basel). 2023 Mar 2;12(5):1144. doi: 10.3390/plants12051144 (PMC10005704; doi:10.3390/plants12051144)
Supplement: Supplementary file 1 [file plants-12-01144-s001.zip › Suppl_Fig_S4_sNMF.pdf]

Wagner et al. 2023

**Supplement Fig. S4 sNMF**

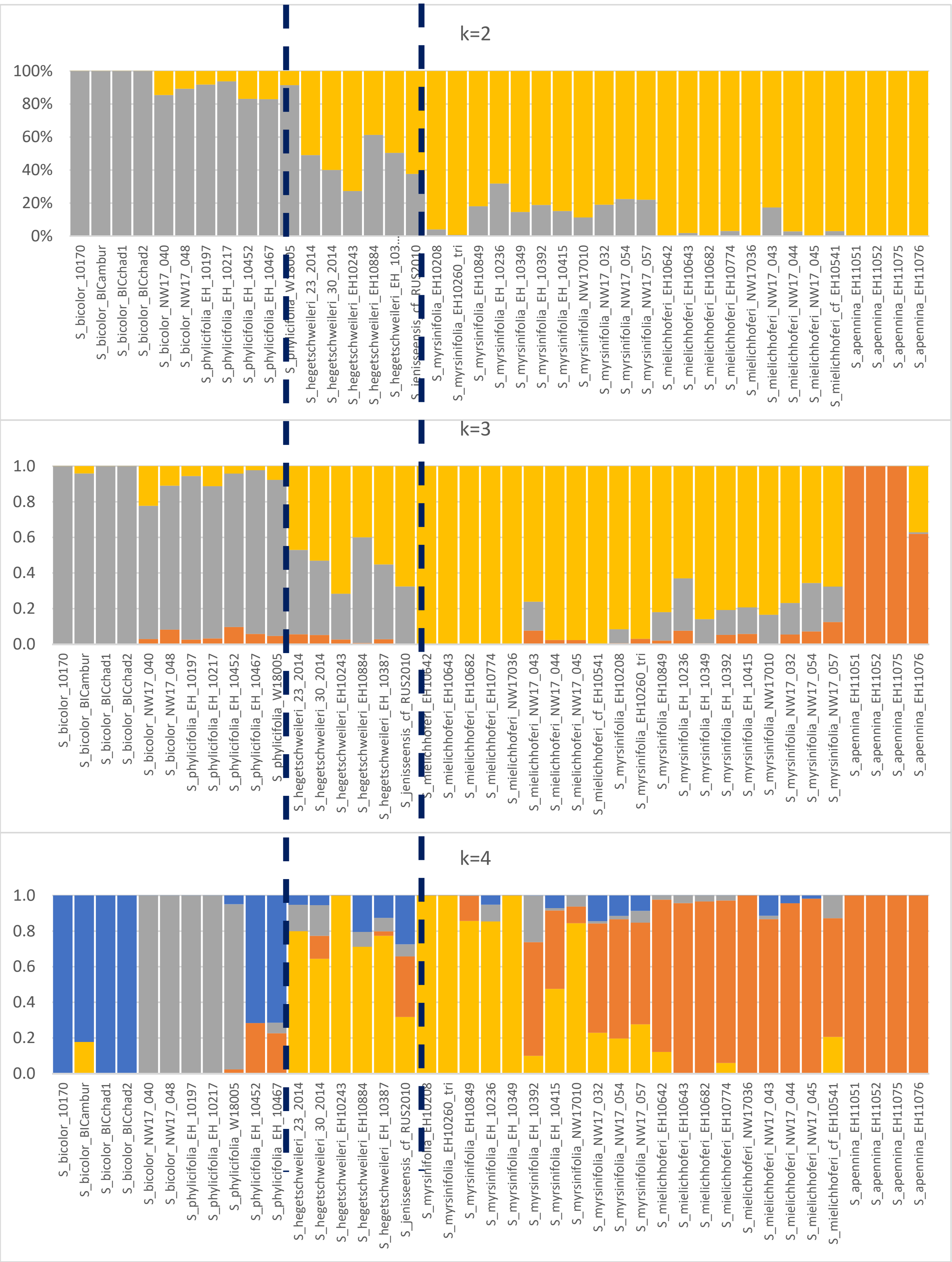


**Fig S4 a Genetic structure analysis with sNMF** including all hexaploid species based on 49,754 RAD loci. Displayed are barplots for three different k values. Colors represent genetic co-ancestry. For k=2, individuals grouping in the clade "*Nigricantes*" are shown in yellow and *Phylicifoliae* in grey. Each bar represents one sample, the respective ID is indicated below each bar

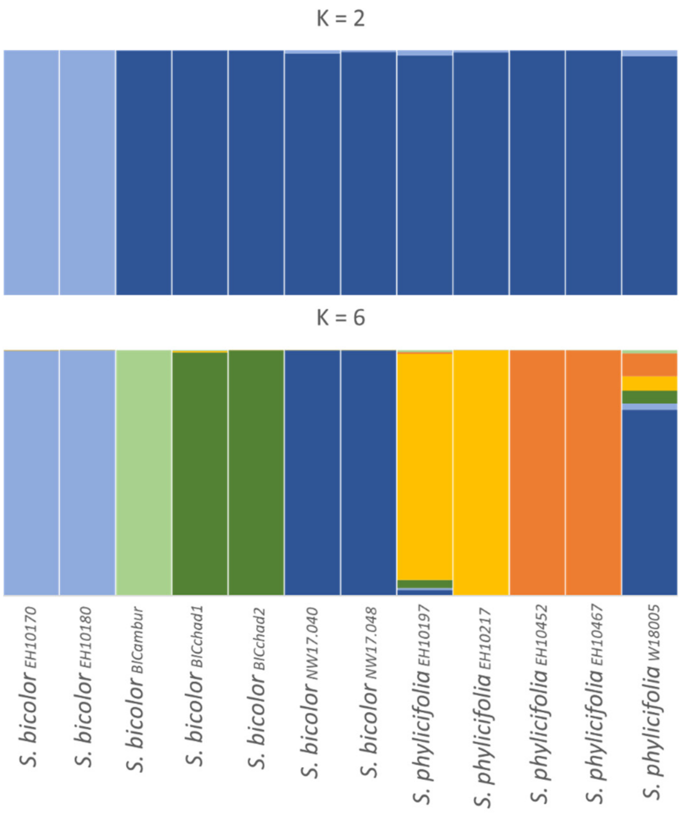


**Fig S4 b.** Barplot representing the results of the genetic structure analysis with sNMF for *S. bicolor* and *S. phylicifolia* for k=2 and k=6 based on 27,010 RAD loci. The genetic structure is indicated by colors. Each bar represents one sample, the respective ID is indicated below each bar.

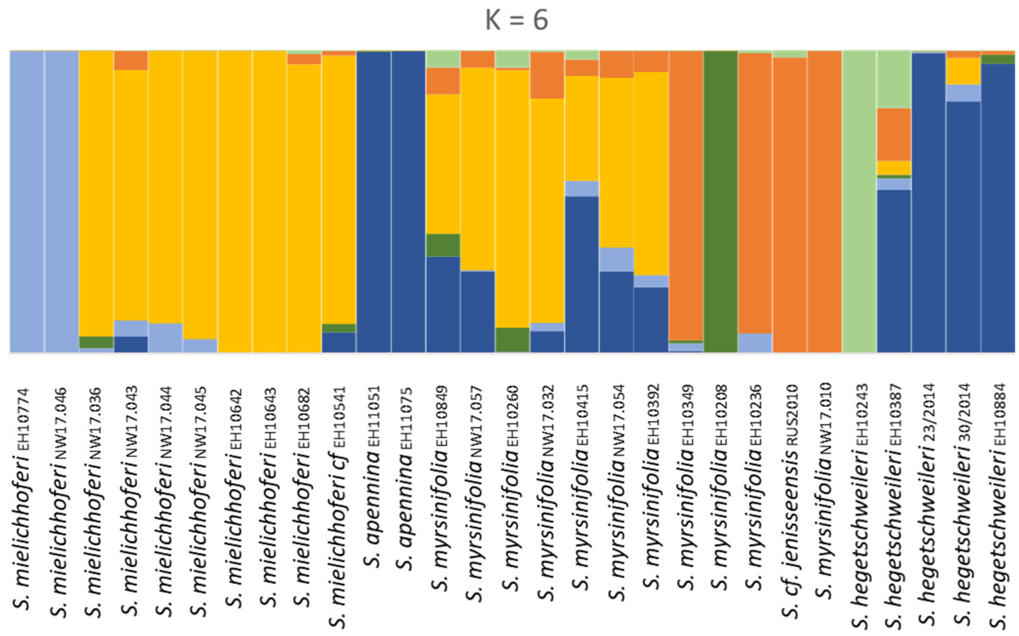


**Fig S4 c**. Barplot representing the results of the genetic structure analysis with sNMF for samples of *section Nigricantes* for k=6 based on 8,164 shared RAD loci. The genetic structure is indicated by colours. Each bar represents one sample, the respective ID is indicated below each bar.
