# Supplementary material for: Insights into the Taxonomically Challenging Hexaploid Alpine Shrub Willows of Salix Sections Phylicifoliae and Nigricantes (Salicaceae)
Source: Plants (Basel). 2023 Mar 2;12(5):1144. doi: 10.3390/plants12051144 (PMC10005704; doi:10.3390/plants12051144)
Supplement: Supplementary file 1 [file plants-12-01144-s001.zip › Suppl_Fig_S5_spetroscopy_results.pdf]

Supplement Fig S5 – results for infrared spectroscopy data of subgroups

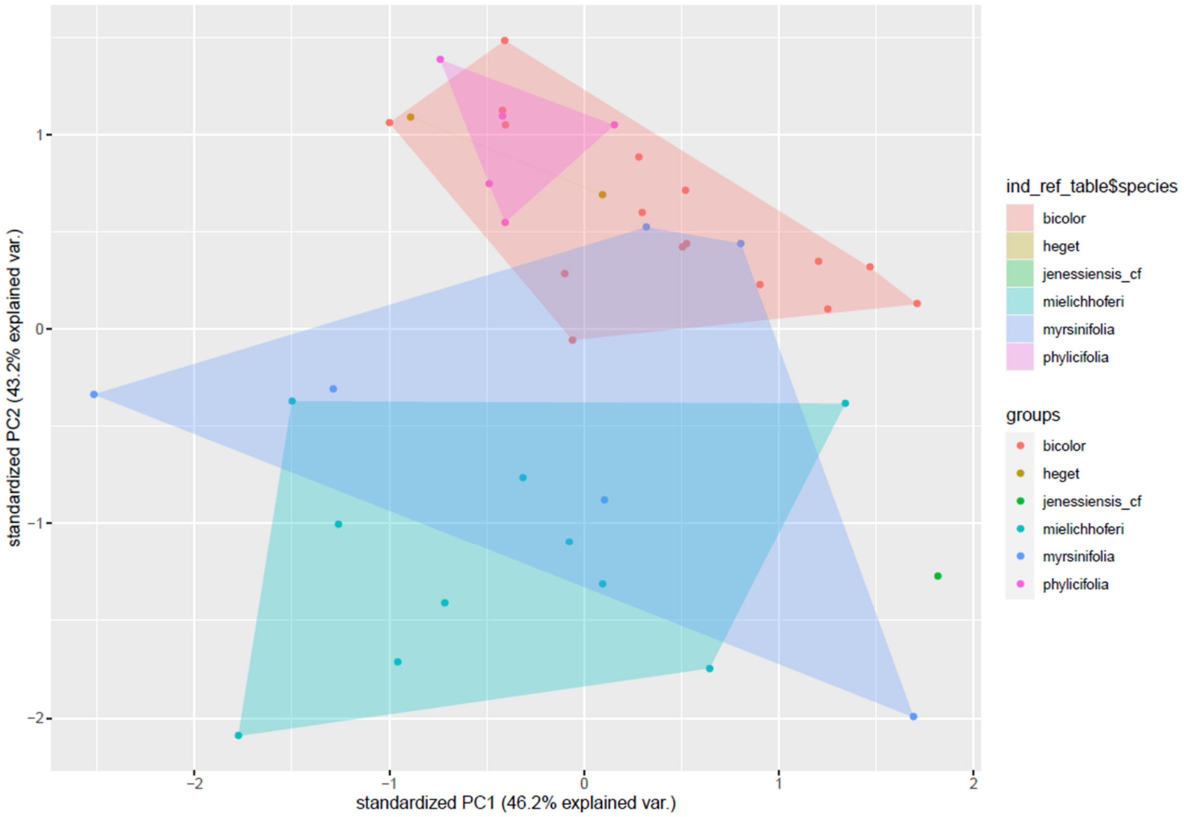

Fig S5 a. PCA of spectral data analyses of all hexaploid species separating *S. bicolor* and *S. phylicifolia.* The individual samples are colour coded according to the legend on the right.

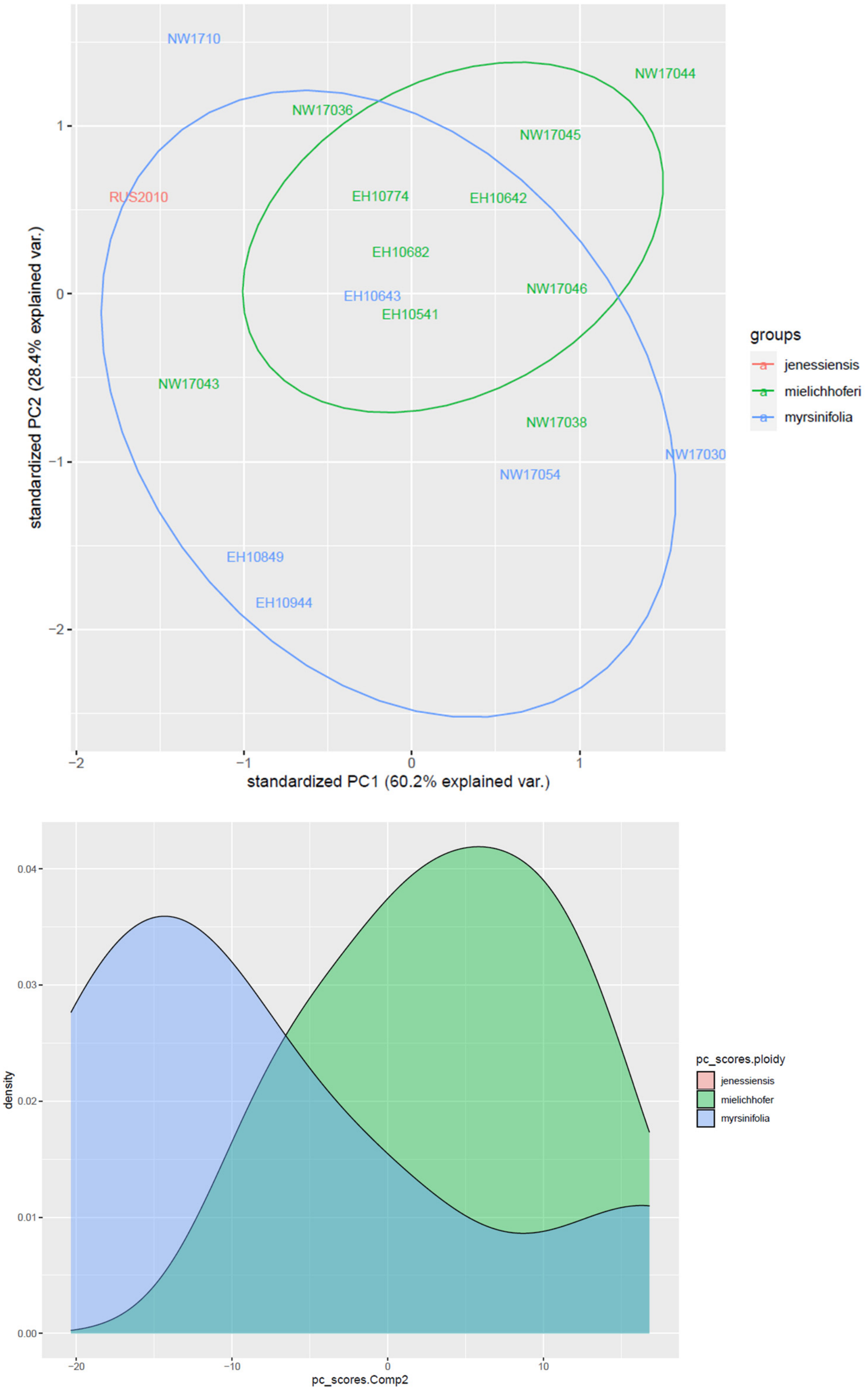

Fig. S5 b. Section *Nigricantes*. PCA and density plot based on infra-red spectroscopy analysed by species assignment. Up to eighteen spectra were used per sample, three adaxial and three abaxial measurements per leaf for three leaves per sample. After filtering the final dataset included 303 spectra on 17 samples of *S. myrsinifolia*, *S. mielichhoferi* and one sample of *S. jenessiensis*. The sample IDs are shown and the assignment to species are colour coded.

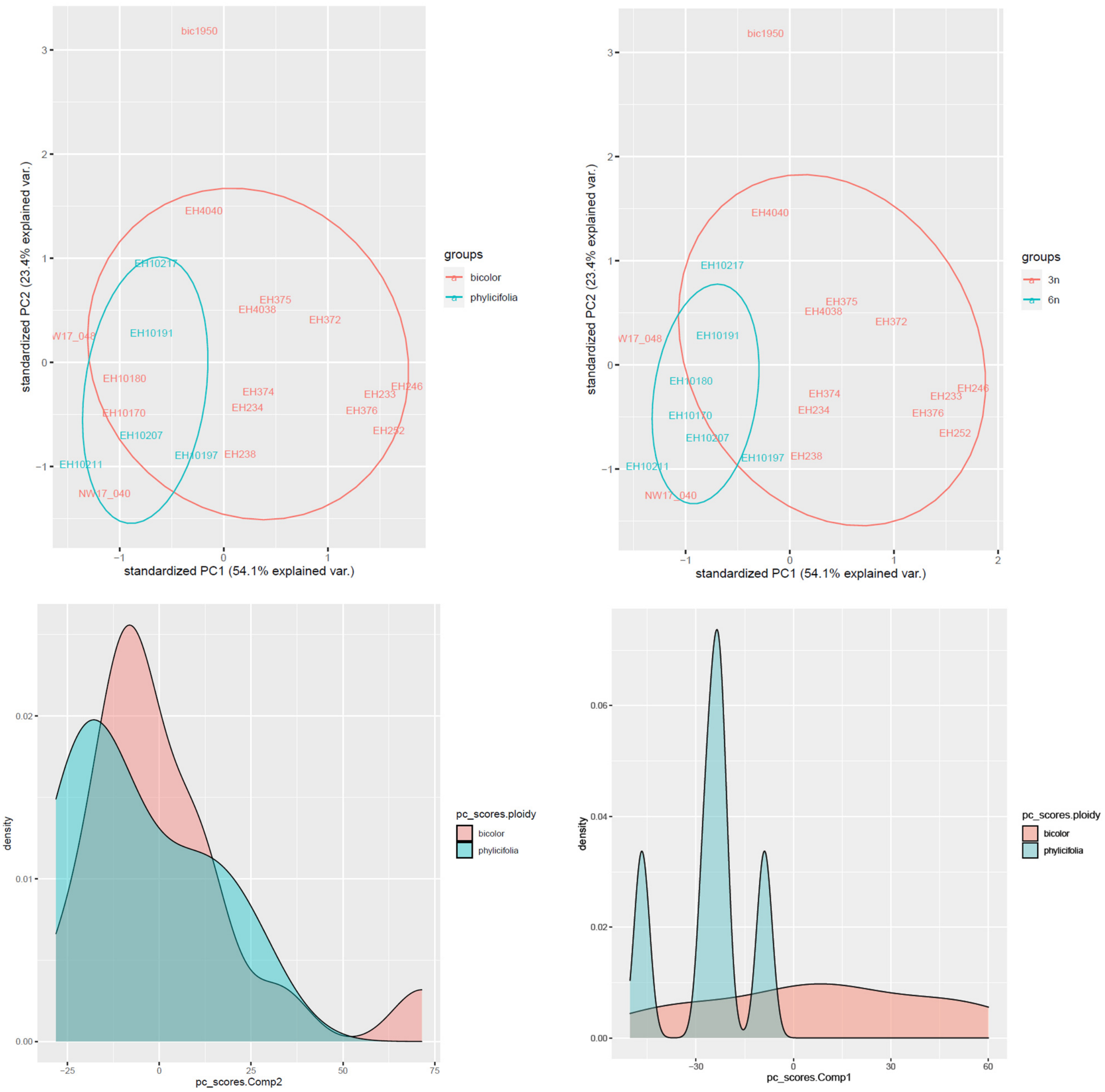

Fig. S5 c. Section *Phylicifoliae*. PCA based on infra-red spectroscopy analysed by species assignment. Up to eighteen spectra were used per sample, three adaxial and three abaxial measurements per leaf for three leaves per sample. After filtering the final dataset included 372 spectra on 23 samples of *S. bicolor*, *S. hegetschweileri* and *S. phylicifolia*. The sample IDs are shown and the assignment to species are colour coded.

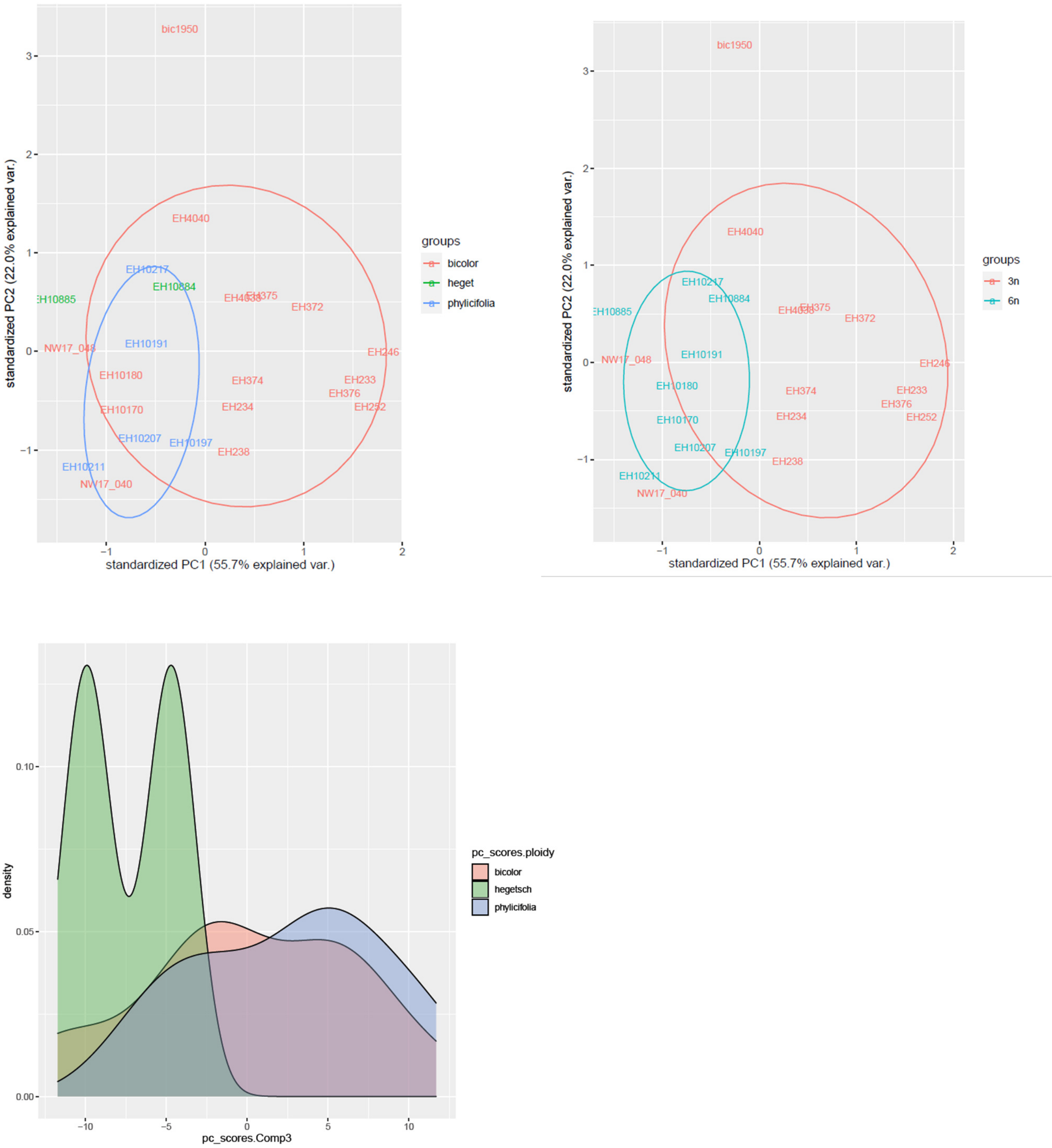

Fig. S5 d. Section *Phylicifoliae incl S. hegetschweileri*. PCA based on infra-red spectroscopy analysed by species assignment. Up to eighteen spectra were used per sample, three adaxial and three abaxial measurements per leaf for three leaves per sample. After filtering the final dataset included 372 spectra on 23 samples of *S. bicolor*, *S. hegetschweileri* and *S. phylicifolia*. The sample IDs are shown and the assignment to species are colour coded.
