# Supplementary material for: Insights into the Taxonomically Challenging Hexaploid Alpine Shrub Willows of Salix Sections Phylicifoliae and Nigricantes (Salicaceae)
Source: Plants (Basel). 2023 Mar 2;12(5):1144. doi: 10.3390/plants12051144 (PMC10005704; doi:10.3390/plants12051144)
Supplement: Supplementary file 1 [file plants-12-01144-s001.zip › Suppl_Fig_S6_PCA_3 taxa.pdf]

Supplement Fig S6 – PCA using five morphological characters, three taxa

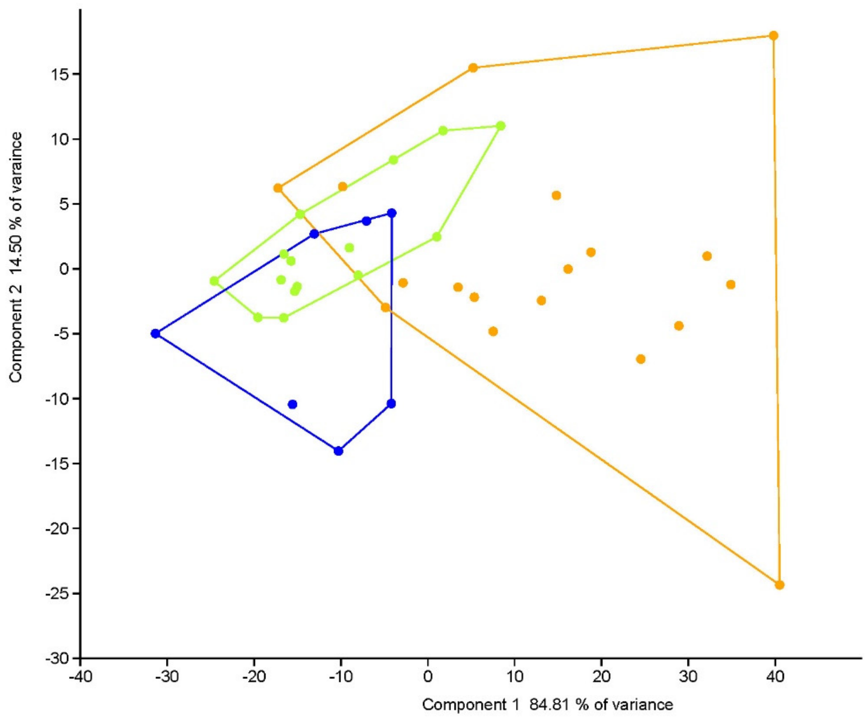

Fig S6 PCA using five morphological characters plotted with convex hull, colors set for three taxa: *S. bicolor* (green), *S. phylicifolia* (blue), *S. hegetschweileri* (orange).

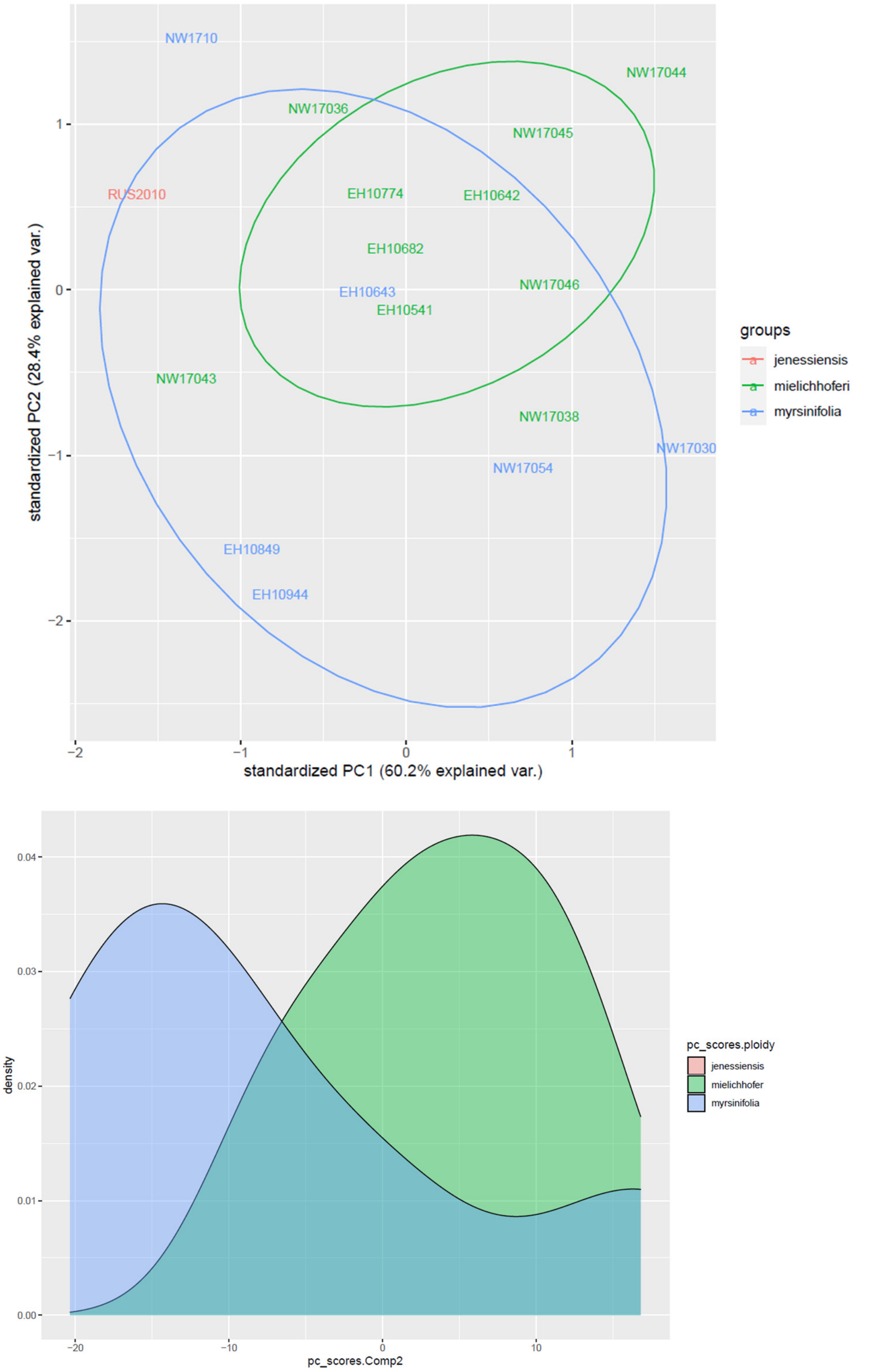

Fig. S5 b. Section *Nigricantes*. PCA and density plot based on infra-red spectroscopy analysed by species assignment. Up to eighteen spectra were used per sample, three adaxial and three abaxial measurements per leaf for three leaves per sample. After filtering the final dataset included 303 spectra on 17 samples of *S. myrsinifolia*, *S. mielichhoferi* and one sample of *S. jenessiensis*. The sample IDs are shown and the assignment to species are colour coded.

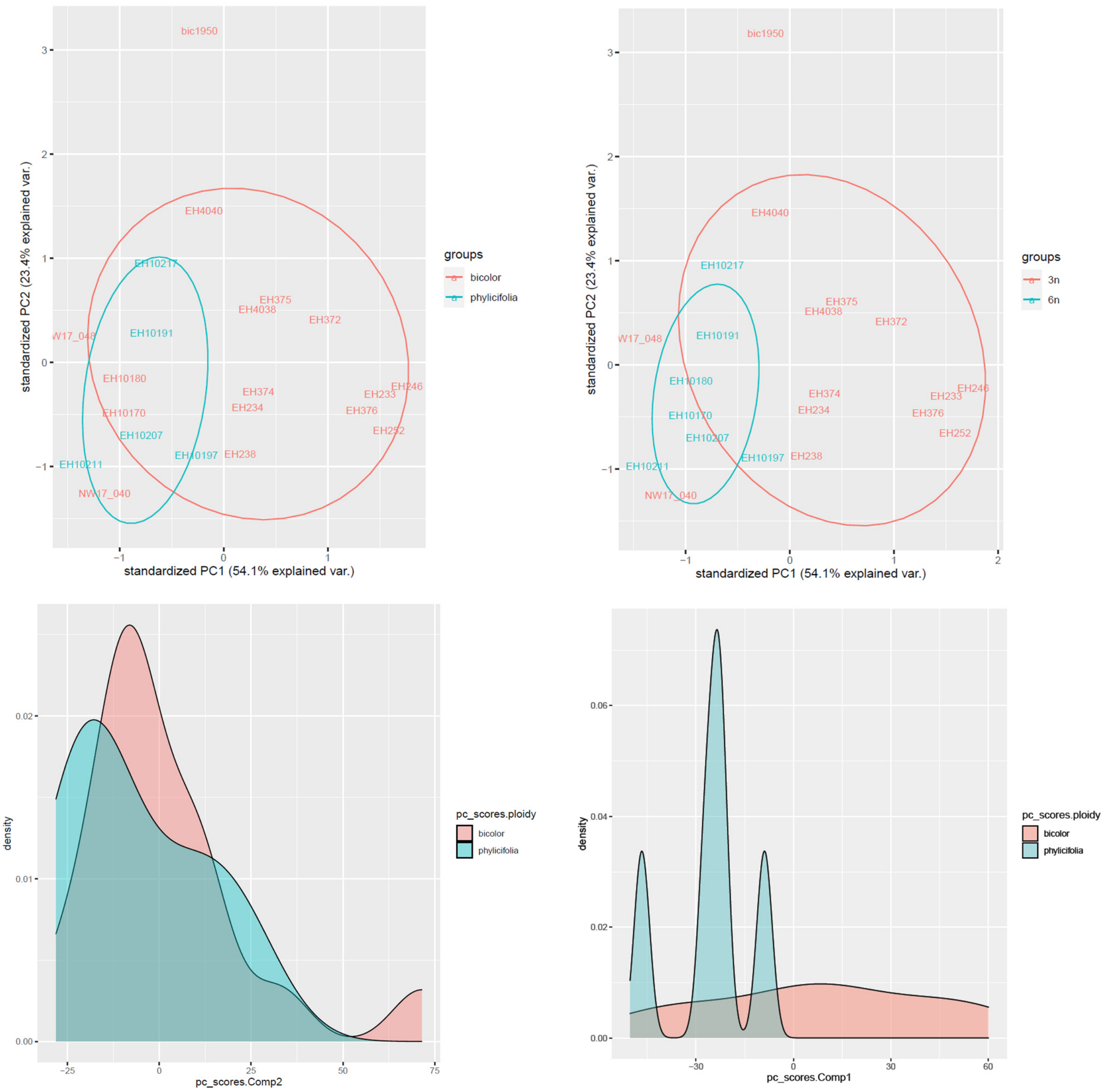

Fig. S5 c. Section *Phylicifoliae*. PCA based on infra-red spectroscopy analysed by species assignment. Up to eighteen spectra were used per sample, three adaxial and three abaxial measurements per leaf for three leaves per sample. After filtering the final dataset included 372 spectra on 23 samples of *S. bicolor*, *S. hegetschweileri* and *S. phylicifolia*. The sample IDs are shown and the assignment to species are colour coded.

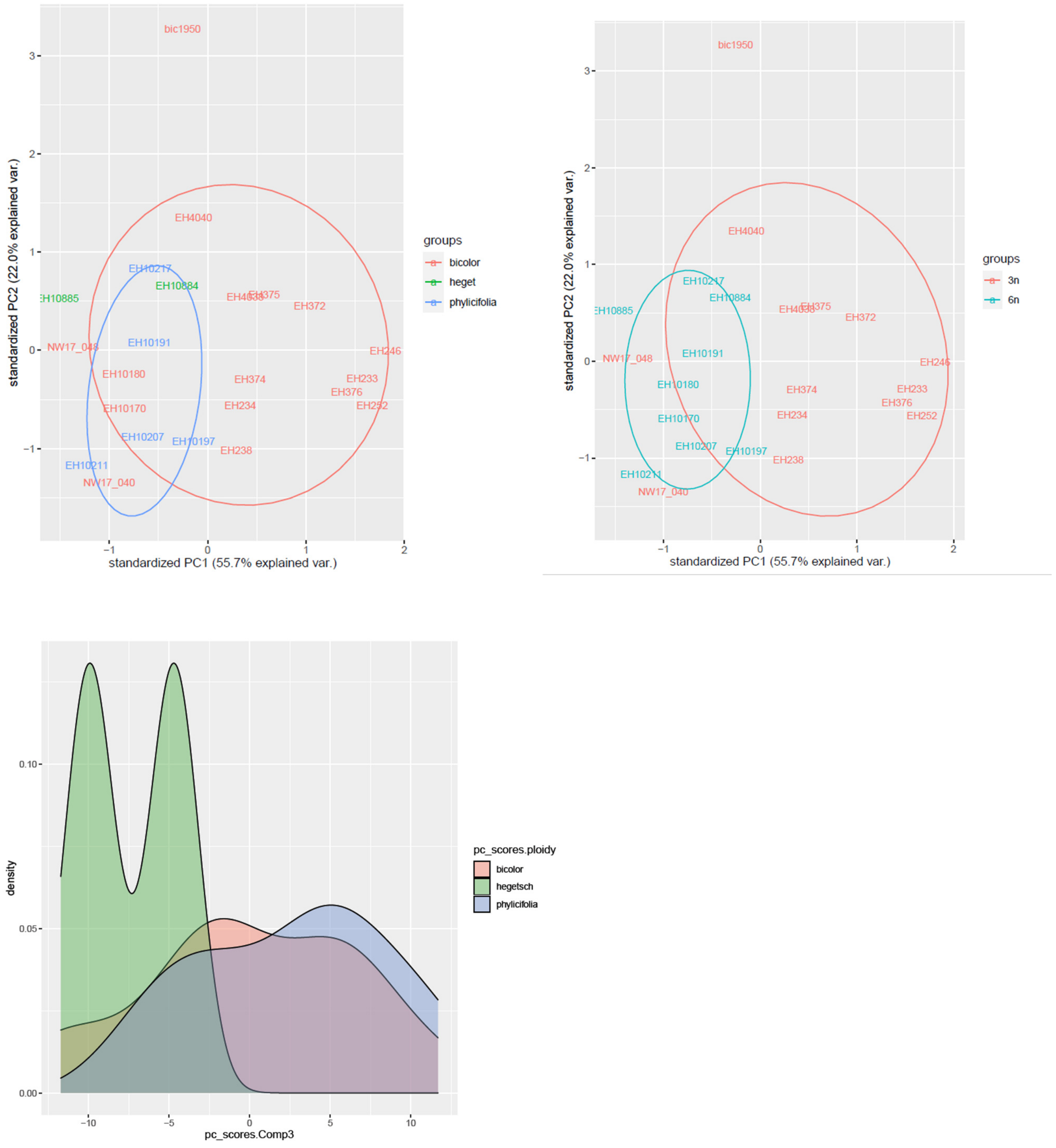

Fig. S5 d. Section *Phylicifoliae incl S. hegetschweileri.* PCA based on infra-red spectroscopy analysed by species assignment. Up to eighteen spectra were used per sample, three adaxial and three abaxial measurements per leaf for three leaves per sample. After filtering the final dataset included 372 spectra on 23 samples of *S. bicolor*, *S. hegetschweileri* and *S. phylicifolia*. The sample IDs are shown and the assignment to species are colour coded.
